# The role of tumor model in magnetic targeting of magnetosomes and ultramagnetic liposomes

**DOI:** 10.1038/s41598-023-28914-4

**Published:** 2023-02-08

**Authors:** Alberto Curcio, Jose Efrain Perez, Sandra Prévéral, Alexandre Fromain, Coralie Genevois, Aude Michel, Aurore Van de Walle, Yoann Lalatonne, Damien Faivre, Christine Ménager, Claire Wilhelm

**Affiliations:** 1 Laboratoire Physico Chimie Curie, PCC, CNRS UMR168, Institut Curie, Sorbonne University, PSL University, 75005 Paris, France; 2 Aix-Marseille University (AMU), French Alternative Energies and Atomic Energy Commission (CEA), French National Center for Scientific Research (CNRS), UMR7265 Institute of Biosciences and Biotechnologies of Aix-Marseille (BIAM), 13108 Saint-Paul-lez-Durance, France; 3 TBM Core, UAR 3427, INSERM US 005, University of Bordeaux, 33000 Bordeaux, France; 4 Laboratoire Physicochimie des Électrolytes et Nanosystèmes Interfaciaux, CNRS, Sorbonne Université, Phenix, 75005 Paris, France; 5 Université Sorbonne Paris Nord, Université Paris Cité, Laboratory for Vascular Translational Science, LVTS, INSERM, UMR 1148, Bobigny, F-93017 France; 6 Département de Biophysique et de Médecine Nucléaire, Assistance Publique-Hôpitaux de Paris, Hôpital Avicenne F- 93009, Bobigny, France

**Keywords:** Nanoparticles, Cancer models

## Abstract

The combined passive and active targeting of tumoral tissue remains an active and relevant cancer research field. Here, we exploit the properties of two highly magnetic nanomaterials, magnetosomes and ultramagnetic liposomes, in order to magnetically target prostate adenocarcinoma tumors, implanted orthotopically or subcutaneously, to take into account the role of tumor vascularization in the targeting efficiency. Analysis of organ biodistribution in vivo revealed that, for all conditions, both nanomaterials accumulate mostly in the liver and spleen, with an overall low tumor retention. However, both nanomaterials were more readily identified in orthotopic tumors, reflecting their higher tumor vascularization. Additionally, a 2- and 3-fold increase in nanomaterial accumulation was achieved with magnetic targeting. In summary, ultramagnetic nanomaterials show promise mostly in the targeting of highly-vascularized orthotopic murine tumor models.

## Introduction

Magnetic nanoparticles have made headway in a wide-ranging number of biomedical applications due to intrinsic properties that enable and make them suitable for their use in this field^1,2^. These include functional coatings to improve targetability, as well as a high biocompatibility in part provided by possible biodegradation and incorporation within the iron internal metabolic pathway of the organism^3^. They can be tailored as drug^4^ or gene carriers^5^, and they can be manipulated or excited by external magnetic sources for therapy (i.e. as in magnetic hyperthermia^6–8^) or regenerative medicine applications such as tissue engineering^9,10^. Within this scope, their responsiveness to magnetic field gradients has been introduced in the field of magnetic targeting, that aims to direct a magnetic carrier to the desired disease or tumoral site using an external or implanted magnet in order to enhance the therapeutic dose effect directly in situ, and thus attenuating potential systemic effects^11–14^. Typically, the nanoparticles are administered via systemic injection and then moved through the bloodstream or tissue by a magnetic gradient until they reach the area of interest.

Initial magnetic targeting formulations tested in vivo consisted of 1–2 µm microspheres composed of 10–20 nm magnetite nanoparticles and doxorubicin embedded in an albumin matrix^15–17^, with the first clinical trial taking place years later^18^. There, a ferrofluid consisting of 100 nm iron nanoparticles was successfully directed to the tumor site with a 50% efficacy. Later clinical trials significantly improved directed targeting rates^19^ as well as treated tumor fractions^20^. Nevertheless, limitations still hinder a full clinical applicability: nanocarriers accumulate in vital organs such as the liver and spleen after the typical intravenous injection^21^, and have a limited blood life before clearance^22^. As they additionally must overcome the multiple constraints of the tumor microenvironment, the low penetrability of nanocarriers into deep tissue is another limitation^23^. On the other hand, the unique vascularization profile of cancerous tumors renders them more permeable to drug penetration and accumulation, a phenomena deemed as the enhanced permeability and retention effect (EPR)^24^. Despite this inherently positive vascularization profile for therapy, the EPR effect offers a low increase in nanocarrier targeting to tumor sites, at a 2-fold increase compared to normal organs^25^. Moreover, the EPR effect appears to be more significant in small tumor models, such as the typical ones used in in vivo experiments, and its influence additionally varies depending on the type of tumoral tissue^26^.

Magnetic targeting enhances to a certain extent the accumulation of nanoparticles in comparison to passive targeting (i.e. the EPR effect), although the percentage of the total injected dose accumulated in target tumors remains low^27^. It has also been recently evidenced that the distinct vascularization parameters (microvessel density, vessel pore size cutoff and vessel diameter) of different types of tumors likely play a role in the efficiency of magnetic nanoparticle accumulation after magnetic targeting^28^. In order to improve this process magnetic targeting, nanocarriers must be tailored in terms of magnetic loading, hydrodynamic size and coating in order to maximize magnetic response while achieving an adequate systemic response and longer blood circulation half-times. For instance, a combination of higher magnetic loading and high magnetic field strength can achieve a more efficient targeting^29^. Magnetic liposomes^30^ have been tested for such a purpose in several in vitro^31^ and in vivo^32^ models, generally subcutaneous tumor models, resulting in an improved therapeutic efficiency when the liposomes were co-vectorized with a chemotherapeutic drug^33,34^. Among highly magnetic theranostic materials are the naturally synthesized magnetosomes^35,36^, which have only recently been tested for in vivo magnetic targeting^37,38^. In addition, the role of tumor model, and especially orthotopic versus subcutaneous tumors, is yet to be investigated in relation with magnetic targeting feasibility and efficiency. Finally, quantification of the targeting effect often involves magnetic resonance imaging, whereas magnetometric quantifications are a robust alternative that offer a direct measure of the amount of magnetic nanomaterials reaching the tumor, in percent of the injected dose, and relative to liver and spleen contents.

Here, our objectives were threefold. We first aimed at determining if magnetic targeting is an effective strategy on cancer models. Second, we selected two types of magnetic carriers that can be utilized according to their high magnetic content. Third, we tested whether this targeting was more effective in orthotopic (OC) or subcutaneous (SC) murine prostate tumor models^32^. We selected ultramagnetic liposomes and magnetosomes as highly magnetic nanomaterials. We systematically quantified the magnetic contents in liver, spleen and tumors by magnetometry, as well as the amount of total iron by elemental analysis, for the two tumor models, the two nanomaterials, and without or upon magnet application (eight groups total, with 4–7 mice per group). Lastly, we correlated the magnetometric measurements with histological and electron microscopy imaging of the three organs (liver, spleen and tumor) showing an enhanced effect for both type of nanoparticles, yet only for OT models.

## Results

**Figure 1:**
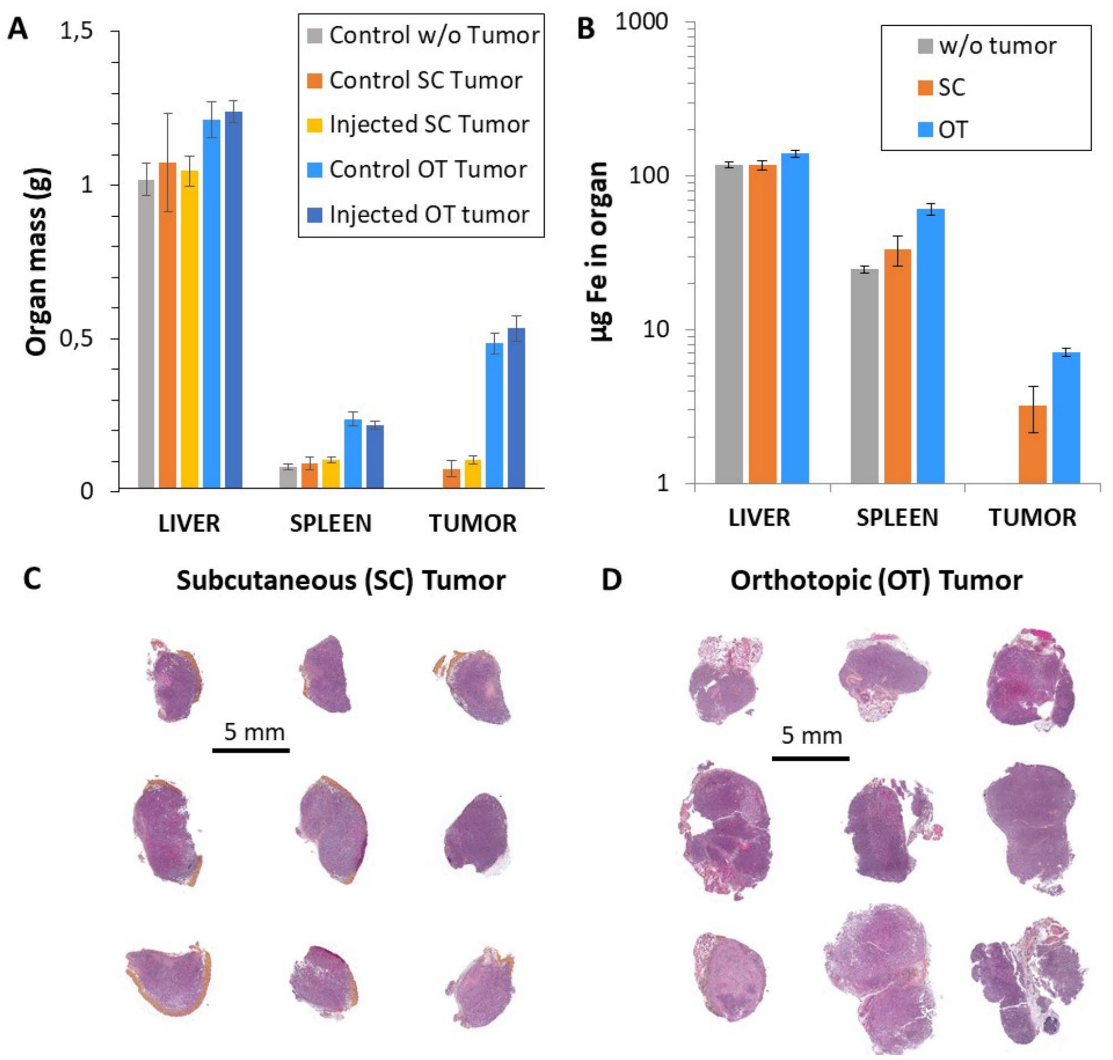
Subcutaneous (SC) versus orthotopic (OT) tumor models. (**A**) Organ mass in grams for liver, spleen and tumor 4 and 7 days after injection for OT and SC models, respectively. (**B**) Iron content (µg Fe) in liver, spleen and tumor of the control mice for the two induced models (*n* = 6 for SC; *n* = 5 for OT). (**C**) and (**D**) Histological slides of SC and OT tumor models, respectively.

Prostate cancer cells (RM-1) were injected either within the prostate or in the posterior right leg in order generate OT or SC tumors, respectively. As RM1 tumors are relatively fast in growth, mature tumors can be obtained within 4 and 7 days after injection for OT and SC tumors, respectively^39^. The SC and OT groups consisted of a total of 29 and 30 mice, respectively. Non-injected control mice were analyzed in parallel (*n* = 6 for SC; *n* = 5 for OT). Results of mass quantification for liver, spleen and tumors are shown in Fig. 1A. Overall, there is an increase in total organ and tumor mass for OT tumors. This is additionally observable in the histological slides of the two tumor models (Fig. 1C,D). Figure 1B additionally shows the quantification of the total iron content for the same organs by inductively coupled plasma spectrometry (ICP). Results are in the range of 100 µg for the liver, between 20 and 50 µg for the spleen, and between 2 and 5 µg in the tumor. The high values, in order of 0.1, 0.2 and 0.2–0.3 g_Fe_ per g of organ, for liver, spleen and tumor, respectively, logically reflect the natural abundancy of iron in the organism and its importance in tissue growth.

**Figure 2:**
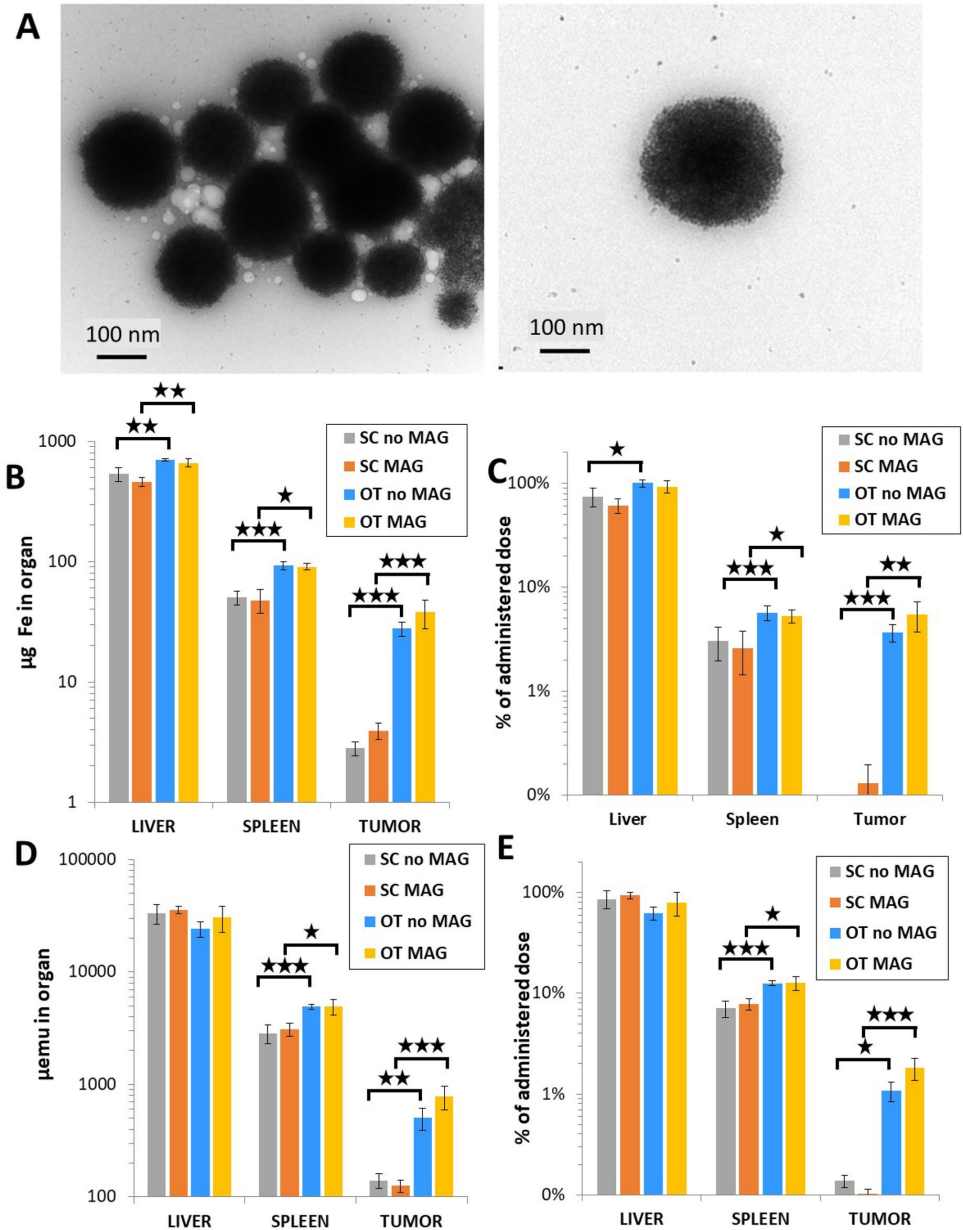
Quantification in organs after ultramagnetic liposomes intravenous administration. (**A**) Transmission electron microscopy micrographs showing the structure of the ultramagnetic liposomes. (**B**) and (**C**) Mass of iron and percentage of the administered total mass of iron, respectively, measured in the liver, spleen and the two tumor models with and without magnet application. (**D**) and (**E**) Magnetic moment (expressed in µemu) and percentage of the administered dose in terms of magnetic moment, respectively, measured under the same conditions. The percentage of the administered dose was calculated as the total content in each organ subtracted by the same for control, normalized by the dose injected (558 µg of iron/mouse) for total iron content (**D**) and for emu (E). *p < 0.05, **p < 0.01 and ***p < 0.001.

Next, Fig. 2 introduces the impact of magnetic targeting using ultramagnetic liposomes in the SC (*n* = 14 mice) and OT tumor models (*n* = 17 mice). The average diameter of the liposomes is 200 nm, and they are composed of an encapsulated core with a high content of maghemite (γ-Fe_2_O_3_) nanoparticles of around 9 nm in diameter (Fig. 2A). The liposomal and encapsulated nanoparticles size distribution quantifications are shown in Supplementary Information Fig. 1. The liposome diameter is within the recommended size limit for long circulation after systemic injection, while assuring at the same time a high magnetic responsivity^40^. A dose of 558 µg_Fe_ per mouse ([Fe] = 100 mM) was administered intravenously (see Materials and methods section) after tumor maturation. For the magnetic targeting group mice (*n* = 7 for both tumor models), the magnet was held in place for two hours immediately after injection. Figure 2B–D show the iron mass quantification for liver, spleen and each of the induced tumor, from total amount of iron quantification (ICP, Fig. 2B) and from magnetic iron quantification (magnetometry, Fig. 2D), with the percentage of the administered dose delivered to each organ shown in Fig. 2C,E, respectively.

In comparison with the endogenous liver iron content of around 100 µg (Fig. 1D), the total amount of iron after ultramagnetic liposome injection with or without targeting was in the range of 600–700 µg. For the spleen, a similar increase in the range of 30–40 µg was observed. For the SC tumor group, the magnetic moment measured in tumors was very low, close to the detection limit, making the measurements unreliable. This probably explains why a magnetic targeting effect was detected from the quantification of total amount of iron by ICP (20–30 µg increase), but not by magnetometry, with no significant difference between the magnet and no magnet groups.

The magnetic signature of iron oxide nanoparticles is an asset in biodistribution studies, allowing the use of unique magnetization characterization tools. Thus, we then proceeded to quantify by vibrating sample magnetometer the specific magnetic fingerprint of the injected liposomes in the mice. The advantage of this quantification is that it excludes the endogenous, non-magnetic iron present in each organ of interest, yielding a more robust measurement of the injected dose reaching each area of interest. The results, expressed in magnetic field moment units, show a similar trend (Fig. 2D) as the amount of total iron. Given the magnetic moment of the ultramagnetic liposomes, estimated at 67 emu/g, we calculated the proportion of injected dose in terms of magnetic moment, shown in Fig. 2E.

In contrast, for the OT tumor models, an approximate 2-fold increase of the administered dose can be found upon magnet application (from around 1 to 2% of the administered dose).

**Figure 3:**
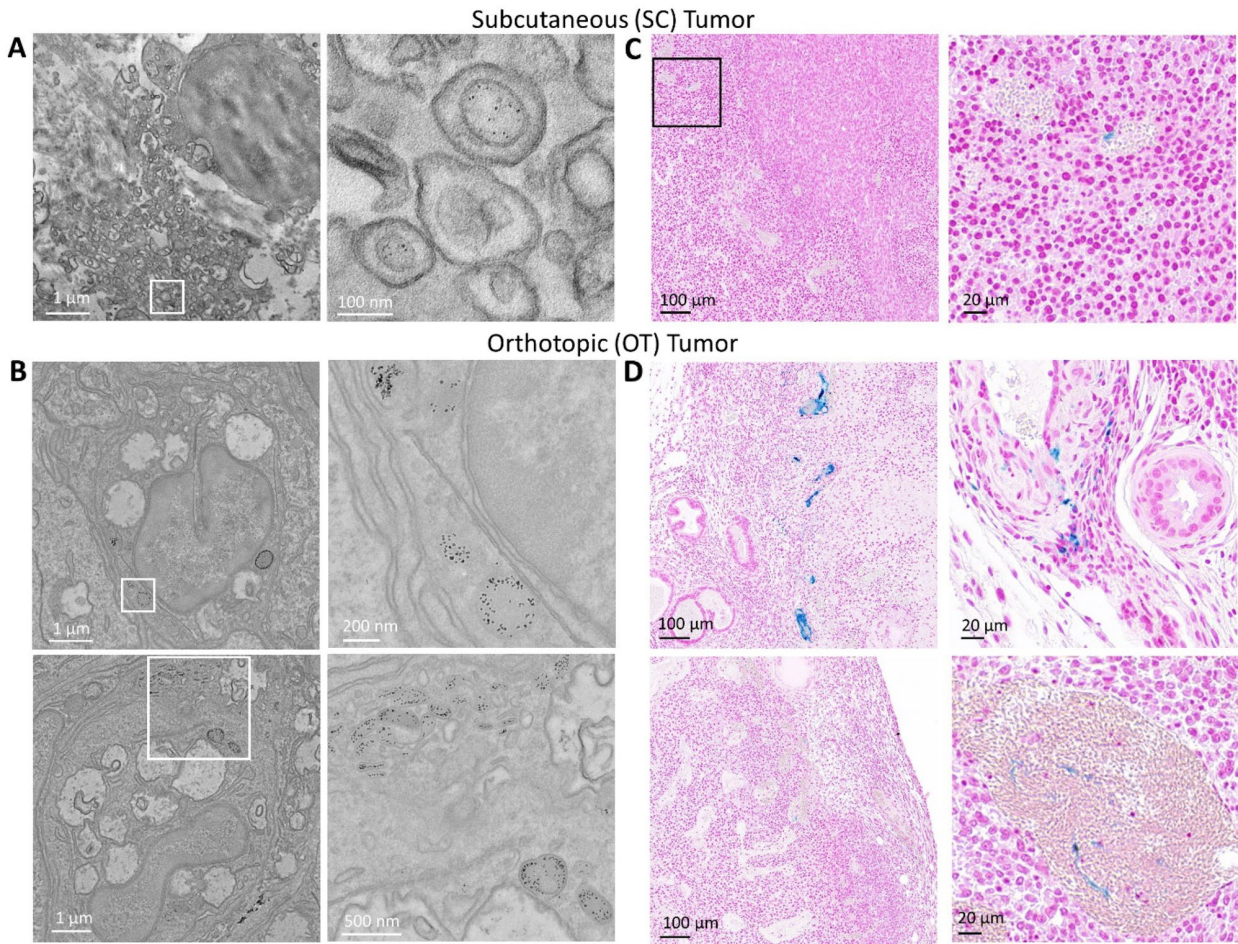
Tumor imaging after ultramagnetic liposomes intravenous administration and magnetic targeting. (**A**) and (**B**) Transmission electron microscope imaging of SC and OT tumors, respectively. (**C**) and (**D**) Histological Prussian Blue tissue staining in SC and OT tumors, respectively. Blue staining denotes the accumulation of ferric iron. Some vessels with iron accumulation within can be observed in OT tumors.

The accumulation of liposomes in both tumor models following magnetic targeting is shown in Fig. 3. Transmission electron microscopy and Prussian Blue histological tissue staining shows a low accumulation of nanoparticles in SC tumors (Fig. 3A,C). On the other hand, nanoparticles were readily identifiable in OT tumors, and were even observed in vessels and within single cancer cells (Fig. 3B,D). Supplementary images of both tumor models show a similar trend (Supplementary Information Figs. 2, 3 and 4). Scarcely any nanoparticles were found for both tumor tissue models when no magnetic targeting was applied (Supplementary Information Figs. 5 and 6).

**Figure 4:**
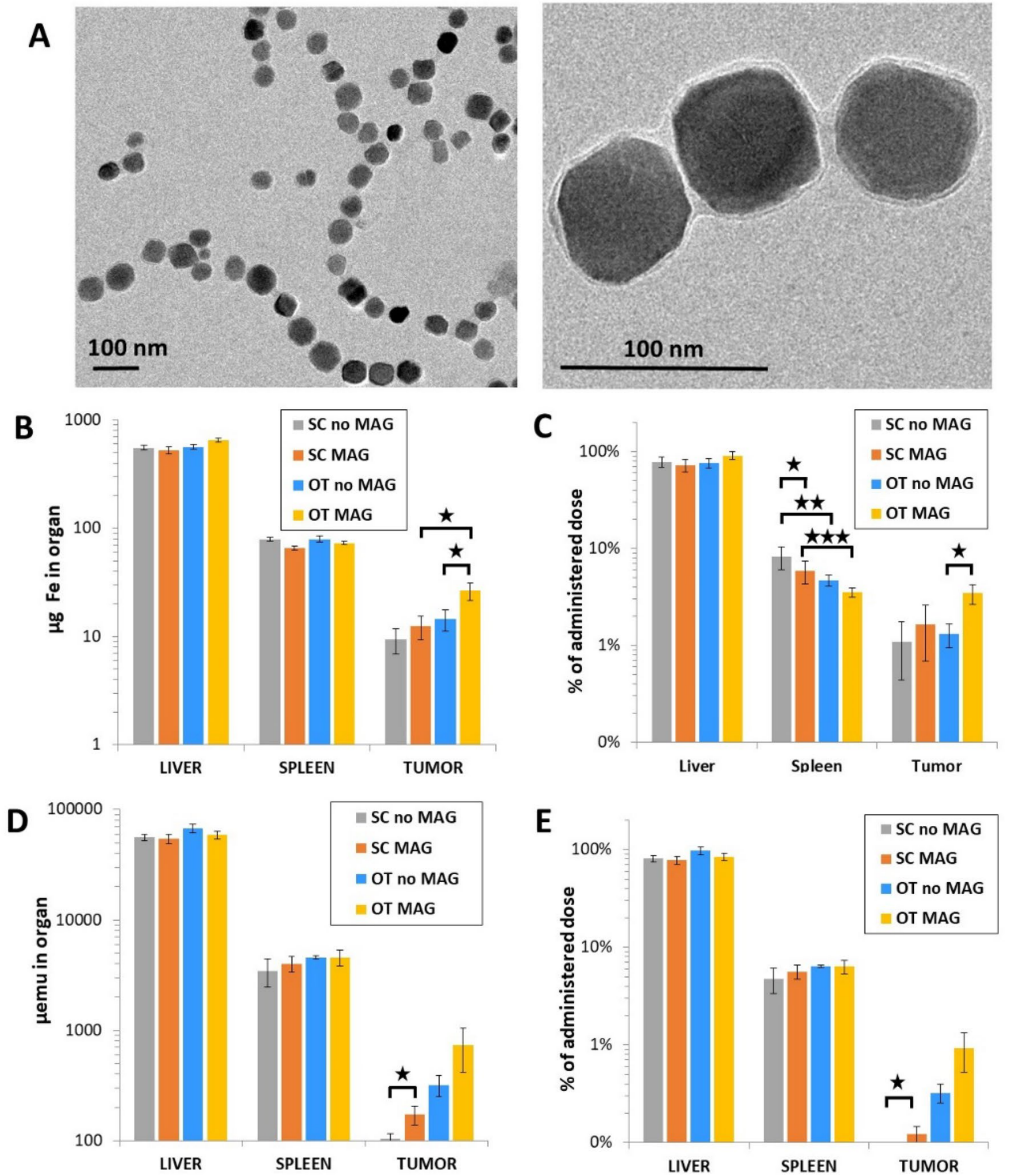
Quantification in organs after magnetosome intravenous administration. (**A**) Transmission electron microscopy imaging of magnetosomes. (**B**) and (**C**) Mass of iron and percentage of the administered total mass of iron, respectively, measured in the liver, spleen and the two tumor models with and without magnet application. (**D**) and (**E**) Magnetic moment and percentage of the administered dose in terms of magnetic moment, respectively, measured under the same conditions. *p < 0.05, **p < 0.01 and ***p < 0.001.

The same biodistribution analyses were performed with magnetosomes. Figure 4 shows the iron mass quantification and magnetometry analysis for both SC and OT tumor models (mice: *n* = 9 for the SC group, of which *n* = 5 with magnetic targeting; *n* = 8 for the OT group, of which *n* = 4 for magnetic targeting). The magnetosomes (Fig. 4A), of an average diameter of 50 nm (Supplementary Fig. 1), possess a magnetite core resulting in a high magnetization of 8 emu/g. The mass of iron administered was the same as the one used with liposomes, of 558 µg_Fe_ per mouse, and remarkably, the total liver iron mass quantification shows a consistent 600 µg. The overall iron mass found in the spleen was 30 µg higher for the magnetosomes (Fig. 4B) than for the liposomes (Fig. 2B). In the case of SC tumors, the total iron mass was found to be 4-fold higher after magnetosome injection in comparison with the liposomes, yet it was found to be slightly lower for magnetosomes in OT tumors. A very similar trend is thus reflected in the percentage of the administered dose delivered to each organ (Fig. 4C), at around 80% and 5% in average for liver and spleen, respectively.

The magnetometry analysis for the magnetosome distribution is shown in Fig. 4D,E. The accumulated percentage of the injected dose in the SC tumors is minimal, with or without magnet application. By contrast, magnetic targeting enhances the retention of magnetosomes in OT tumors 3-fold, from 0.3 to 1% of the total injected dose. This corresponds to about 700 µemu, roughly 6 µg of iron that are equivalent to an approximate 10 billion magnetosomes in the tumor. The biodistribution of the magnetosomes in terms of magnetic material was on average 85% and 5% of the administered dose found in liver and spleen, respectively.

**Figure 5:**
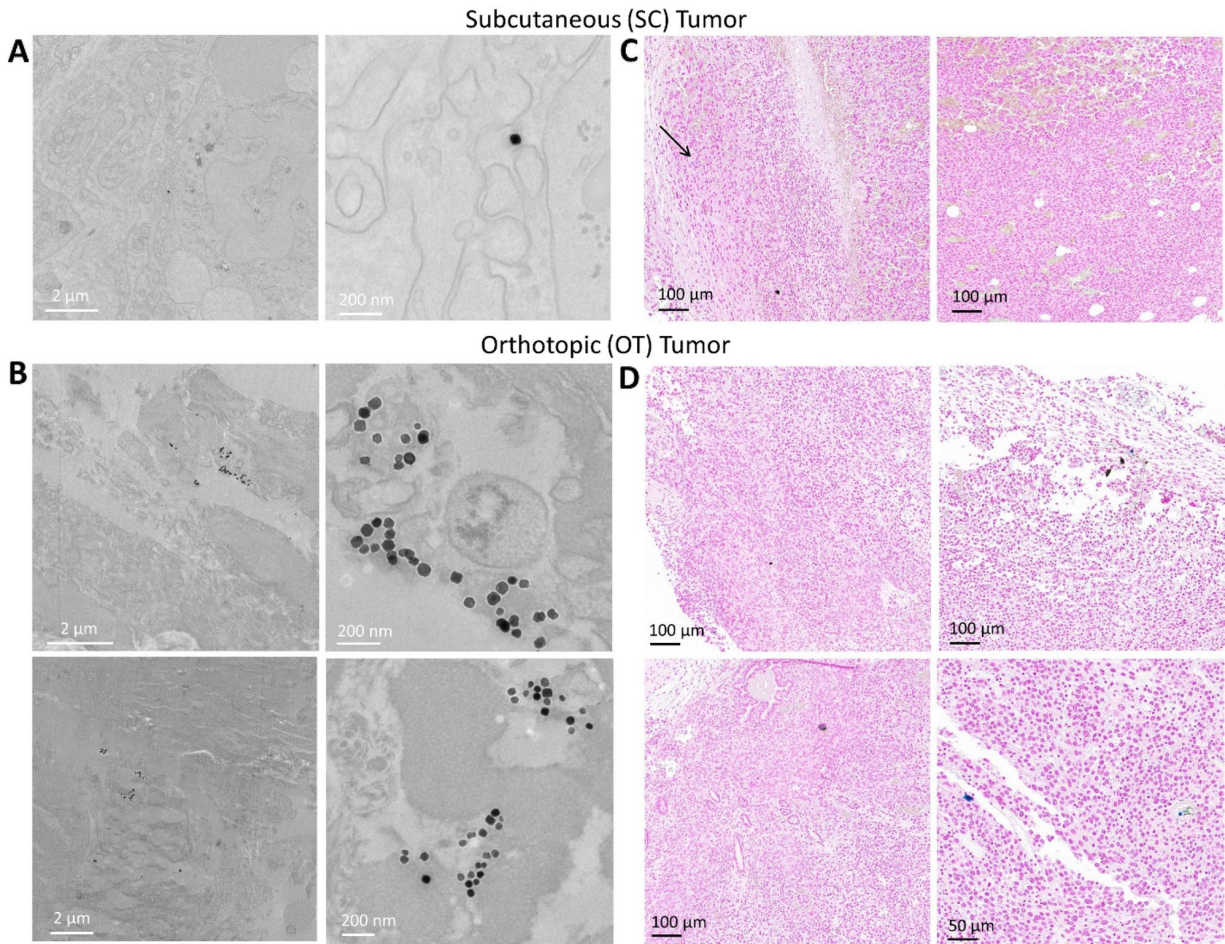
Tumor imaging after magnetosome intravenous administration and magnetic targeting. (**A**) and (**B**) Transmission electron microscope imaging of SC and OT tumors, respectively. (**C**) and (**D**) Histological Prussian Blue tissue staining in SC and OT tumors, respectively.

As expected, it proved difficult to locate magnetosomes in SC tumors by transmission electron microscopy imaging and histological Prussian Blue staining (Fig. 5A,C). Much like the ultramagnetic liposomes, the accumulation of magnetosomes was more evident in OT tumors (Fig. 5B, D). Additional images of magnetosomes in OT models upon magnetic targeting are provided in the Supplementary Information Figs. 7 and 8. Magnetosomes were hard to observe in both SC and OT tumor models that were not exposed to the magnetic force (Supplementary Information Figs. 9 and 10).

Overall, for both ultramagnetic liposomes and magnetosomes in both tumor models, the percentage of area occupied by nanoparticles was largely distributed over the tumor histological section images, as per Prussian Blue staining quantification shown in Supplementary Fig. 11. Liposomes show average values in the range of 0.07% and 0.02% for SC tumors with and without magnetic targeting, respectively, and of 0.4%, and 0.1% for OT tumors under the same respective conditions. Similarly, magnetosomes cover SC tumor areas of 0.09% and 0.01% with and without magnet application, respectively, and of 0.13% and 0.03% in OT tumors under the same conditions.

## Discussion

Here, we evaluated the efficiency of magnetic targeting of either ultramagnetic liposomes or magnetosomes in SC and OT prostate cancer tumor models in vivo. Taken together, the results indicate that OT tumors are more susceptible to retain nanomaterials, probably due to their higher vascularization compared to SC tumors. Indeed, it has been reported that OT prostate cancer tumor models possess higher vascular volume with a wider spatial vascular distribution^41^, which in turn translates to a significantly higher perfusion compared to SC tumors, at around 4-fold^42^.

Without magnetic targeting, more liposomes (1.1% ID/g) concentrate in the OT tumors than magnetosomes (0.3% ID/g), although they are both within the same dose percentage range. Upon magnetic targeting, these values increase to 1.8% and 1% ID/g for liposomes and magnetosomes, respectively. This could make a difference in treatment, with a 2- to 3-fold increase in the quantity of nanomaterials in OT tumors thanks to magnet application, and similar to results obtained in in vivo brain^43^ and colon^29^ tumor models. The %ID/g obtained with magnetic targeting of magnetosomes is also comparable to that recently reported for this kind of nanoparticles after magnetic targeting in breast cancer tumor models^37,38^.

Neverthe less, these values remain in the low range compared to the accumulation in the liver, with over 80% of the injected dose in all scenarios tested. This limitation of the study highlights the feasibility of improving the targeting to the tumor site via specific functionalization of the nanoparticles to ensure longer bioavailability, which has been recently shown to increase nanoparticle tumor accumulation up to 5-fold after magnetic targeting, albeit in a SC tumor model^44^. Similarly, the use of specific cancer targeting molecules has been reported to increase tumor retention up to 13.8% ID/g after magnetic field application^23^. This was additionally demonstrated by Thébault et al., who used High-Intensity Focused Ultrasound to destabilize the lipid bilayer of ultramagnetic liposomes loaded with the vascular disrupting agent CA4P in order to trigger the latter’s local release. This strategy allowed to obtain a significant improvement in tumor volume regression, all the while using a CA4P loading dose 150-fold lower compared to its typically used chemo-therapeutic dose^34^. Overall, the low nanomaterial retention reported in this study could be partly explained by the poor perfusion of the tumor vasculature. Indeed, in the less perfused SC tumors, with or without magnetic targeting, the percentage of the injected dose reaching the tumors are below 0.1% for both ultramagnetic liposomes and magnetosomes, highlighting the inferiority of this model for the studying of drug magnetic targeting.

In addition to tumor permeability, nanoparticle size plays a critical role in the latter’s targeting efficacy, it being more relevant in tumors with inherent low perfusion. For instance, in a multiparametric tumor targeting study using drug-loaded micelles of increasing diameters, it was found that only the 30 nm nanoparticle formulation achieved tumor growth suppression, with a tumor accumulation of 10% ID/g of tissue, and at 2- and 4-fold times higher than the 50 and 100 nm nanoparticle formulations, respectively^45^. Furthermore, this size-dependent nanoparticle efficiency was absent in a high perfusion tumor model. These results are in agreement with those reported by Chan and colleagues, who reported an ideal nanoparticle size of 60 nm in average for improved uptake, arguing that smaller sized nanoparticles are harder to mediate through active targeting, whereas larger ones are cleared faster from the bloodstream^46^. Indeed, while magnetic targeting may help in part to overcome the naturally low perfusion of the tumor, as we report here, it proves insufficient to achieve a significant retention percentage of the initial injected dose.

As previously mentioned, microvessel density is a major contributing factor to the % ID/g of tumor after magnetic targeting^28^. In that study, dose retention percentages between 3 and 5% were achieved after magnetic targeting with magnetic nanocapsules of similar average diameters (200 nm) tested in colon, breast, lung and melanoma tumor models, corresponding to approximately a 2- to 3-fold increase when compared with passive EPR targeting. In the case of prostatic cancer, microvessel density has been estimated at around 13–50 vessels/mm^2,47^^,^^48^, being significantly lower than that of the reported values for colon, breast, lung and melanoma tumors, which range between 100 and 300 vessels/mm^2,28^. Therefore, this markedly low vasculature parameter in prostatic adenocarcinoma could be a primary contributor to the lower %ID/g reported here after magnetic targeting.

## Conclusion

Tumor targeting efficiency of ultramagnetic liposomes and magnetosomes can be improved by 2- and 3-fold upon magnetic field application in an orthotopic prostatic adenocarcinoma murine tumor model. While the overall particle tumor retention remains low, the study highlights the capability of highly magnetically responsive nanomaterials in in vivo biodistribution and tumor targeting studies. The overall percentage of retention can be improved by specific coatings and ligands to render the nanoparticles more readily assimilated by the tumor. Lastly, the combination with drugs could further their potential in cancer therapy.

## Materials and methods

### Magnetic liposome synthesis

The preparation of ultramagnetic liposomes, from magnetic nanoparticle synthesis to liposome encapsulation has been thoroughly described previously^49^. Briefly, an aqueous suspension of maghemite (γ-Fe_2_O_3_) nanoparticles was synthesized following the standard Massart procedure^50^ through alkaline coprecipitation of FeCl_3_ and FeCl_2_ at a 5:3 molar ratio. Superparamagnetic maghemite grains were then obtained by iron nitrate-facilitated oxidation of magnetite. After several washing steps in acetone and ether, nanoparticles were suspended in deionized water and sorted by size by repeated steps of HNO_3_ addition into the suspension followed by magnetic decantation. Citration of magnetic nanoparticles was achieved by heating up the suspension at 80 °C for 30 min after the addition of sodium nitrate. Nanoparticles were then precipitated in acetone and resuspended in deionized water. The volume fraction and average nanoparticle diameter were determined through the fitting of the magnetization values of the nanoparticles using the Langevin’s Law. In this study, nanoparticles with an average size of 9 nm were selected. The nanoparticles possess a polydispersity index of σ = 0.35, volume fraction of nanoparticles in suspension of φ = 1.9% and a specific susceptibility of χ/ϕ = 15.5.

Ultramagnetic liposomes were then prepared via reverse phase evaporation^32^, with the following materials all purchased from Avanti Polar Lipids, Inc.: chloroform solutions of 1,2-dipalmitoyl-sn-glycero-3-phosphocholine (DPPC), 1,2-distearoyl-sn-glycero-3-phosphocholine (DSPC) and 1,2-distearoyl-sn-glycero-3-phosphoethanolamine-N-[(carboxy(polyethylene glycol)2000] (ammonium salt) (DSPE-PEG2000). Chloroform and diethyl ether were purchased from Carlo Erba reagents and VWR, respectively. Thus, a 250 µL 85/10/5% mol mixture of DPPC/DSPC/DSPE-PGE2000 was mixed in chloroform at 3.3 mg/mL and diluted first in chloroform to 1 mL and then in diethyl ether to 4 mL. Then, the previously synthesized magnetic nanoparticles were added into the mixture. The mixed solution was then sonicated for 20 min, resulting in a water–oil emulsion. The organic solvents in the preparation were then evaporated using a rotavapor R210 (Buchi) at 30 °C, and the water-dispersed liposomes were filtered using a 0.4 µm filter, and the non-encapsulated nanoparticles were magnetically sorted out using a NdFeB magnet (0.3 T) during a 12 h period, repeated twice. The resulting ultramagnetic liposomes were resuspended in 5 mM sodium citrate.

### Magnetosome synthesis

Magnetite magnetosomes were biosynthesized using Magnetospirillum magneticum bacterial strain AMB-1 (ATCC 700264) following a previously established procedure^35^. The Venus reporter was inserted in the bacterial lipid membrane under strict genetic control in order to render the membrane trackable by fluorescence. The modified AMB-1 strain was then cultured in a 6 L bioreactor, with cells in the late exponential-phase of growth being harvested by centrifugation (7500 g for 10 min at 4 °C). The cells were resuspended in 150 mL of a purification buffer, consisting of 20 mM HEPES, 1 mM EDTA, 0.9% NaCl and 8% glycerol at a pH of 7.5 and in the presence of a cocktail of proteases. The cells were then disrupted three times using a French press at 1000 PSI at 4 °C, and then left at the same temperature while in contact with a MACSi-MAGtm separator magnet (Miltenyi Biotec) in order to collect the magnetosomes. magnetosomes were resuspended in 45 mL of purification buffer. This magnetic purification was performed five times in purification buffer without anti-proteases, and then five subsequent times in purification buffer without EDTA. After washing, the magnetite magnetosomes were resuspended in purification buffer without EDTA and NaCl, at a concentration of 3 g/L iron.

### Nanoparticle dose quantification

The nanoparticle iron dose was quantified by inductively coupled plasma optical emission spectroscopy (ICP-OES), using a ACROS spectrometer (SPECTRO Analytical Instruments GmbH). The nanoparticles were first degraded in 100 µL of 69% nitric acid, and then diluted several times in 2% nitric acid to reach an iron concentration in the range of 1–200 ppb (µg/L) before analysis.

### In vivo orthotopic and subcutaneous tumor model induction

Balb/c Mice at 6 weeks of age, weighing 25 g on average and free of pathogens were purchased from Charles River Laboratories, France, and subsequently were acclimatized for one week (Animalerie Buffon, Institut Jacques Monod, Paris). Orthotopic tumor generation was done by direct cell injection within the prostate while under anesthesia with 2% isoflurane (Belamont, Nicholas Piramal Limited, London, UK) in air (Supplementary Information Fig. 12). The abdominal muscles were first incised and the seminal glands were pulled back. Then, RM-1 murine prostatic adenocarcinoma cells in isotonic saline solution were injected in the dorsal prostate lobes, at 5 × 10^5^ cells per 10 µL per lobe. Subcutaneous tumors were induced by injection of 2 × 10^6^ cells per 100 µL in isotonic water in the posterior right leg.

### Magnetic nanoparticle injection and in vivo magnetic targeting

Both tumors were allowed to grow for 8 days before nanoparticle injection. A single dose of 558 µg of iron in 100 µL of saline solution ([Fe] = 100 mM) was injected through the tail for both ultramagnetic liposomes and magnetosomes in both orthotopic and subcutaneous tumor models. Control group mice were injected with saline solution. Shortly before nanoparticle injection, mice were immobilized inside an in-house built system consisting of an aeriated tube on which a 6 × 2 mm neodymium magnet (Supermagnete, Germany) was fixed in order to achieve magnetic targeting of the nanoparticles to the tumoral area while under anesthesia (Supplementary Information Fig. 12). The magnet was left in this position during 2 h. Mice were euthanized by cervical dislocation at one day after the treatment and organs were harvested. Control group mice without nanoparticle injection were handled at equal time intervals.

### Histological analysis

The harvested tumors were excised and divided into smaller pieces, and then fixed overnight with phosphate-buffered 10% formalin at pH 7.4. Then, samples were dehydrated with a series of increasing graded ethanol solutions (30–100%), and then embedded in paraffin. Sections of 8 µm thickness were cut and then stained for Perls Prussian Blue (1% potassium ferrocyanide in 1% hydrochloric acid) (Sigma Aldrich) and with hematoxylin–eosin (Sigma Aldrich).

### TEM analysis

Tumor pieces were fixed in 2% glutaraldehyde in 0.1 M cacodylate buffer for 1 h at room temperature. Samples were then contrasted with oolong tea extract 0.5% and post-fixed in 1% osmium tetroxide with 1.5% potassium cyanoferrate. Shortly afterwards, samples were subjected to a series of dehydration steps in graded ethanol solutions (30% to 100%) and then embedded in epoxy resin. 70 nm slices were cut and placed on 200 mesh copper grids and counterstained with lead citrate before analysis with a Hitachi HT 7700 TEM operated at 80 kV (Elexience, France). Sample preparation was carried out in a microwave tissue processor for electron micrsocopy (KOS-Millestone Medical).

### Magnetometry

The amount of iron present in each harvested organ was quantified using a vibrating sample magnetometer (Quantum Design, Versalab), with each sample being measured at 300 K as a function of the external field (0–3 T). Each sample was then frozen in liquid nitrogen, lyophilized and ground to a powder using a mortar and pestle. High field analysis (3 T) provided magnetization at saturation. The recorded saturate magnetization was converted to grams of iron in the organ using the saturate magnetization of the as-synthesized ultramagnetic nanomaterials.

### Statistical analysis

The independent Student’s t-test was used to calculate the significance between two different groups. A minimum of 95% confidence level was considered, with values of *p < 0.05, **p < 0.01 and ***p < 0.001 considered statistically significant.

### Ethical approval

This study is reported in accordance with ARRIVE guidelines. All animal manipulations were carried out under European-community guideline requirements for animal handling. The experiments were registered to the ethics committee with APAFis reference 201905231559578, under the title “Evaluation du ciblage magnétique de nanoparticules d’oxyde de fer dans des modèles tumoraux prostatiques orthotopiques et sous-cutanées”.

### Supplementary Information

Supplementary Information.

## Data Availability

The datasets and images generated during the course of this work are readily available upon reasonable request from the corresponding author.
