# Author Correction: The role of tumor model in magnetic targeting of magnetosomes and ultramagnetic liposomes

**DOI:** 10.1038/s41598-023-40771-9

**Published:** 2023-08-24

**Authors:** Alberto Curcio, Jose Efrain Perez, Sandra Prévéral, Alexandre Fromain, Coralie Genevois, Aude Michel, Aurore Van de Walle, Yoann Lalatonne, Damien Faivre, Christine Ménager, Claire Wilhelm

**Affiliations:** 1 Laboratoire Physico Chimie Curie, PCC, CNRS UMR168, Institut Curie, Sorbonne University, PSL University, 75005 Paris, France; 2 Aix-Marseille University (AMU), French Alternative Energies and Atomic Energy Commission (CEA), French National Center for Scientific Research (CNRS), UMR7265 Institute of Biosciences and Biotechnologies of Aix-Marseille (BIAM), 13108 Saint-Paul-lez-Durance, France; 3 TBM Core, UAR 3427, INSERM US 005, University of Bordeaux, 33000 Bordeaux, France; 4 Laboratoire Physicochimie des Électrolytes et Nanosystèmes Interfaciaux, CNRS, Sorbonne Université, Phenix, 75005 Paris, France; 5 Université Sorbonne Paris Nord, Université Paris Cité, Laboratory for Vascular Translational Science, LVTS, INSERM, UMR 1148, Bobigny, F-93017 France; 6 Département de Biophysique et de Médecine Nucléaire, Assistance Publique-Hôpitaux de Paris, Hôpital Avicenne, F-93009 Bobigny, France

Correction to: *Scientific Reports* 10.1038/s41598-023-28914-4, published online 08 February 2023

The Acknowledgements and Funding sections in the original version of this Article were omitted.

The Acknowledgements section now reads:

“The authors acknowledge the CNanoMat platform of Sorbonne Université Paris Nord for VMS magnetometry, the HistIM platform at Cochin Institute for histology analysis (Maryline Favier), the IPGP multidisciplinary program PARI and Paris–IdF region SESAME Grant no. 12015908 for ICP–AES analyses (Laure Cordier) and the MIMA-2 platform from INRAE, Jouy en Josas, for TEM imaging (Christine Péchoux).”

The Funding section now reads:

“The present work was funded by the European Research Council project ERC-2019-CoG NanoBioMade 865629.”

The original Article has been corrected.

