# Supplementary material for: The role of tumor model in magnetic targeting of magnetosomes and ultramagnetic liposomes
Source: Sci Rep. 2023 Feb 8;13:2278. doi: 10.1038/s41598-023-28914-4 (PMC9908874; doi:10.1038/s41598-023-28914-4)
Supplement: Supplementary file 1 — Supplementary Information. [file 41598_2023_28914_MOESM1_ESM.pdf]

Supplementary Information

The role of tumor model in magnetic targeting of magnetosomes and ultramagnetic liposomes

Alberto Curcio[1+], Jose Efrain Perez[1+], Sandra Prévéral[2], Alexandre Fromain[1], Coralie Genevois[3], Aude Michel[4], Aurore Van de Walle[1], Yoann Lalatonne[5], Damien Faivre[2], Christine Ménager[4] and Claire Wilhelm[1]*

1. Laboratoire Physico Chimie Curie, PCC, CNRS UMR168, Institut Curie, Sorbonne University, PSL University, 75005 Paris, France.

2. Aix-Marseille University (AMU), French Alternative Energies and Atomic Energy Commission (CEA), French National Center for Scientific Research (CNRS), UMR7265 Institute of Biosciences and Biotechnologies of Aix-Marseille (BIAM), Saint-Paul-lez-Durance, 13108 France

3. TBM Core, UAR 3427, INSERM US 005, Univ. Bordeaux, F-33000, Bordeaux, France.

4. Sorbonne Université, CNRS, Laboratoire Physicochimie des Électrolytes et Nanosystèmes Interfaciaux, PHENIX, 75005 Paris, France.

5. Inserm, U1148, Laboratory for Vascular Translational Science, Université Paris 13, Sorbonne Paris Cité, Bobigny F-93017, France.

+ Equal contribution

* corresponding author : claire.wilhelm@curie.fr

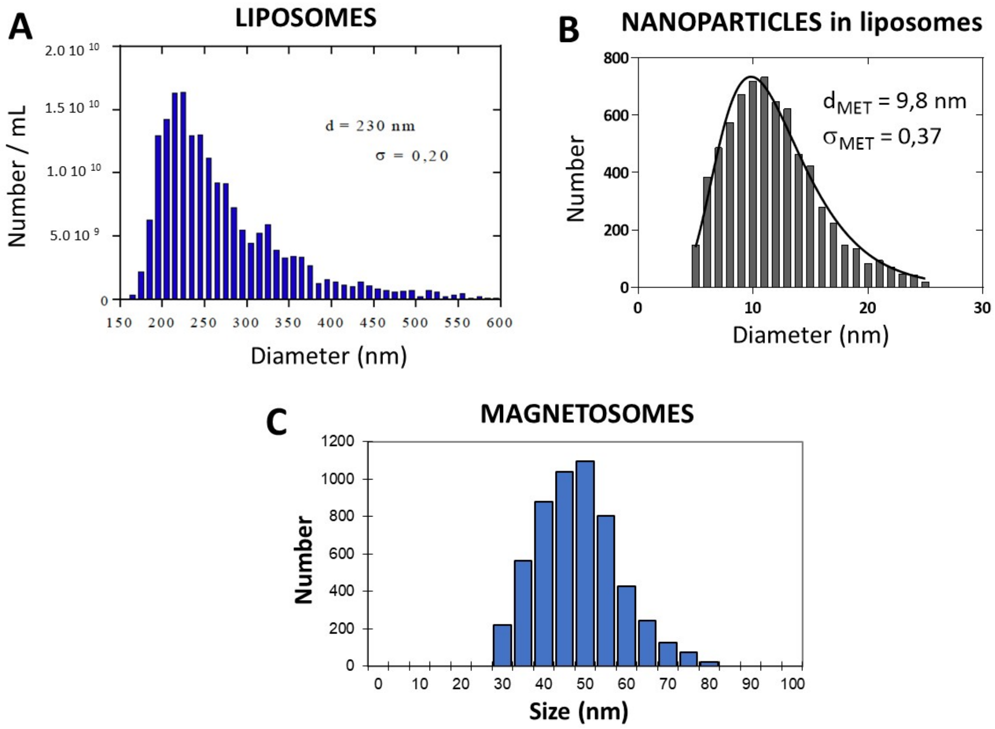

Figure 1. Size distribution of ultramagnetic liposomes and magnetosomes. A) Size distribution of liposomes, measured at the single particle level by Tunable Resistive Pulse Sensing. B) and C) Size distribution of the encapsulated nanoparticles within the liposomes and of magnetosomes, respectively, both quantified from TEM images.

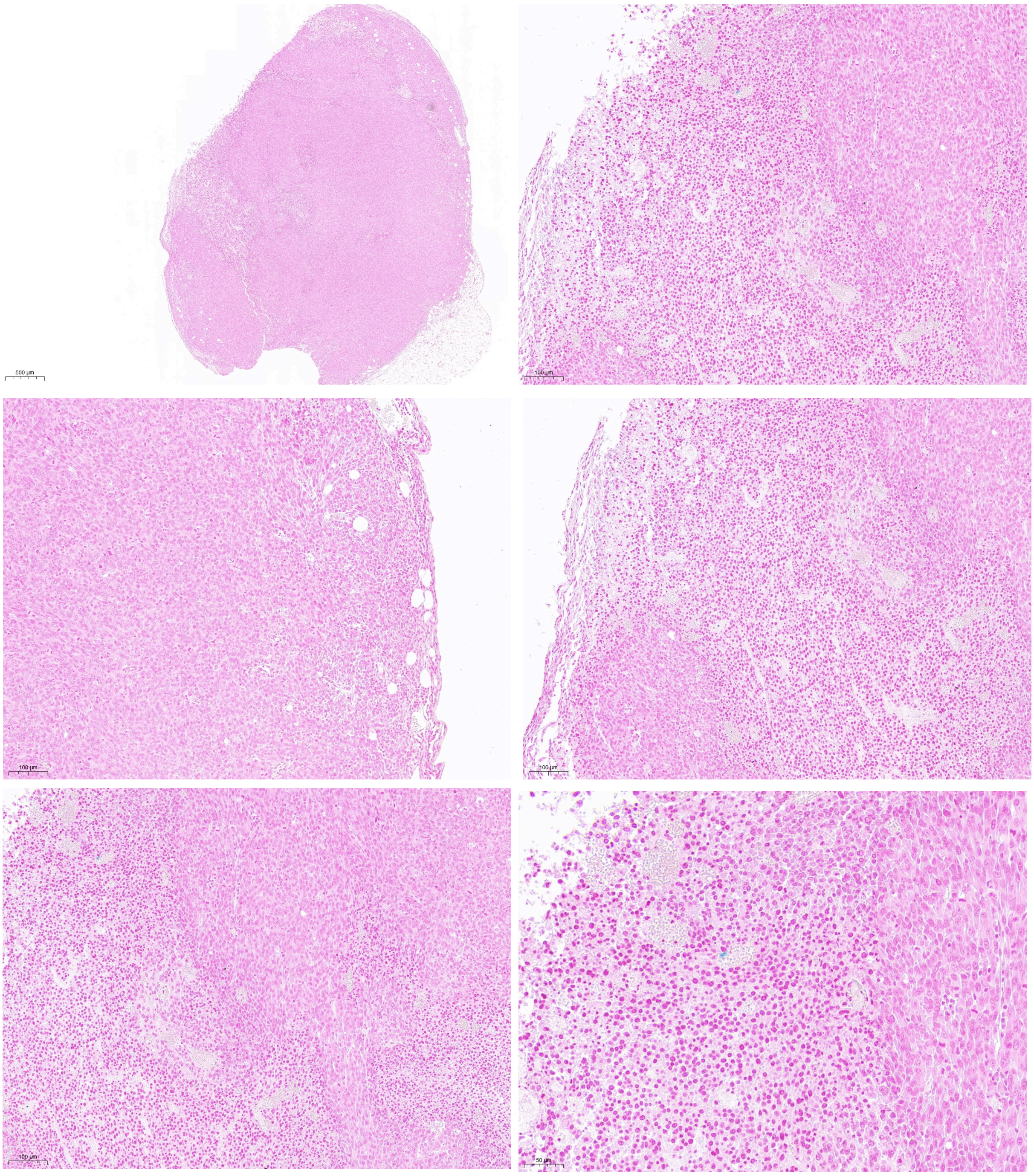

Figure 2. Histological Prussian Blue tissue staining of subcutaneous tumor models after intravenous administration of ultramagnetic liposomes and upon magnet application.

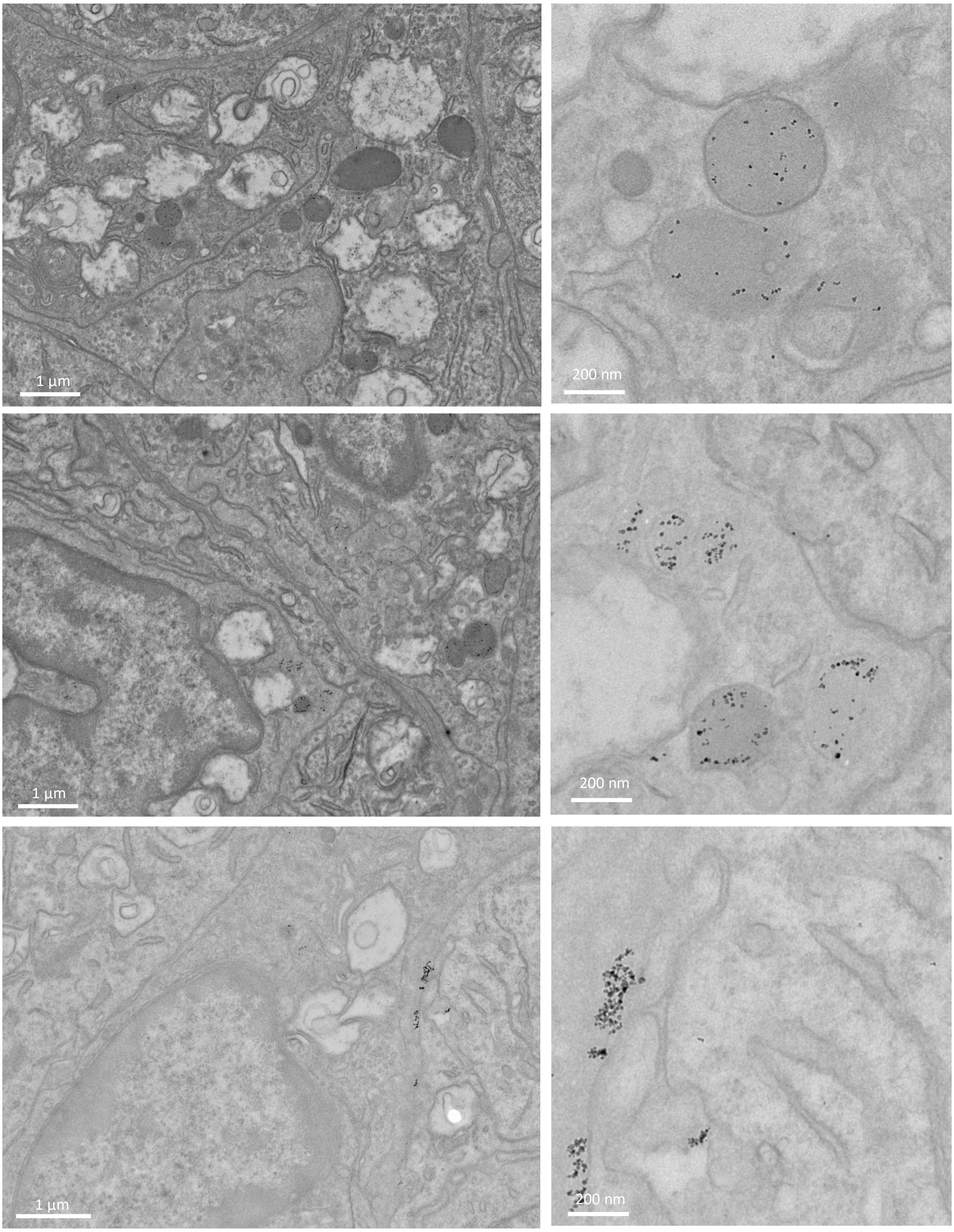

Figure 3. Transmission electron microscope imaging of orthotopic tumor models after intravenous administration of ultramagnetic liposomes and upon magnet application.

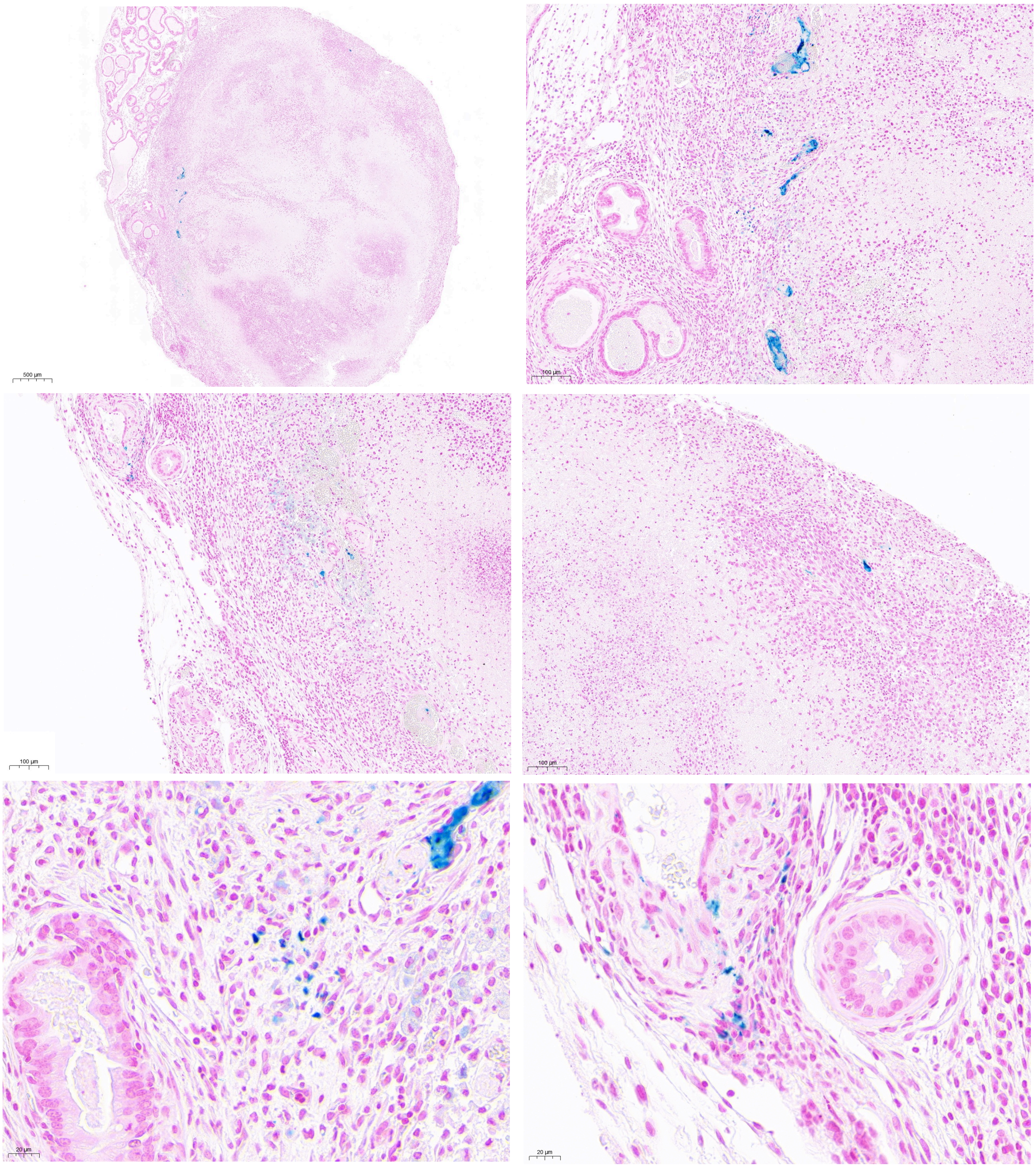

Figure 4. Histological Prussian Blue tissue staining of orthotopic tumor models after intravenous administration of ultramagnetic liposomes and upon magnet application.

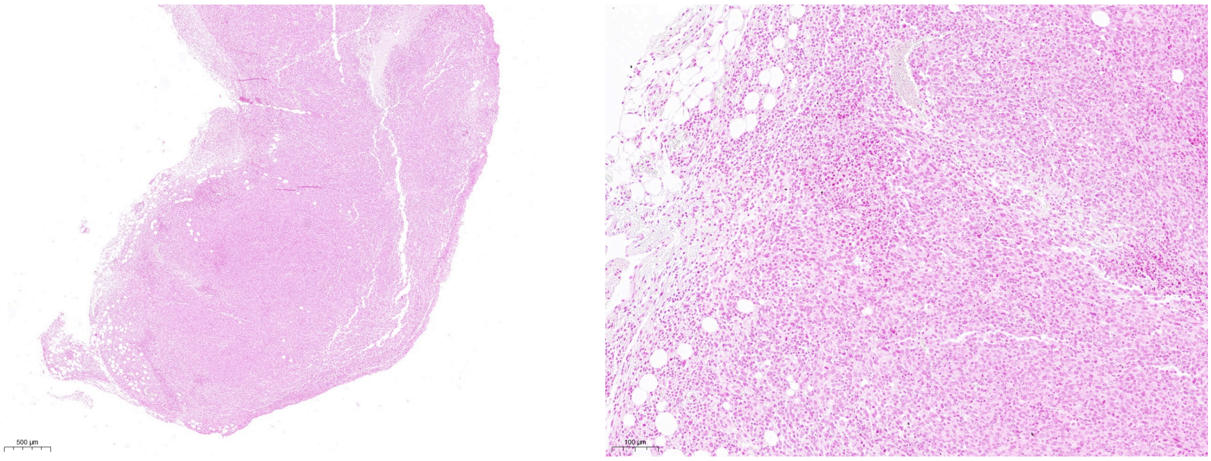

Figure 5. Histological Prussian Blue tissue staining in subcutaneous tumor models after intravenous administration of ultramagnetic liposomes and without magnet application.

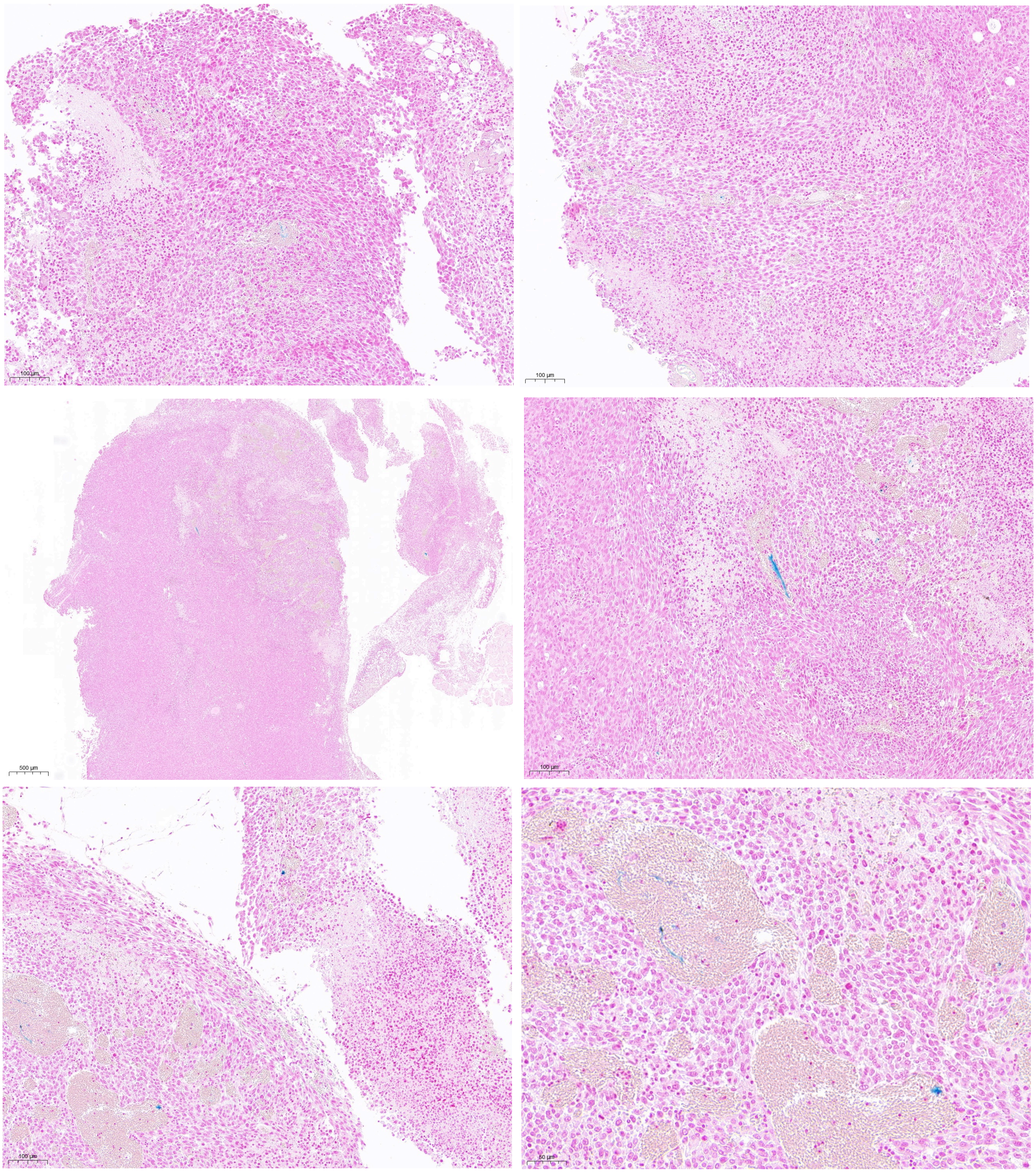

Figure 6. Histological Prussian Blue tissue staining in orthotopic tumor models after intravenous administration of ultramagnetic liposomes and without magnet application.

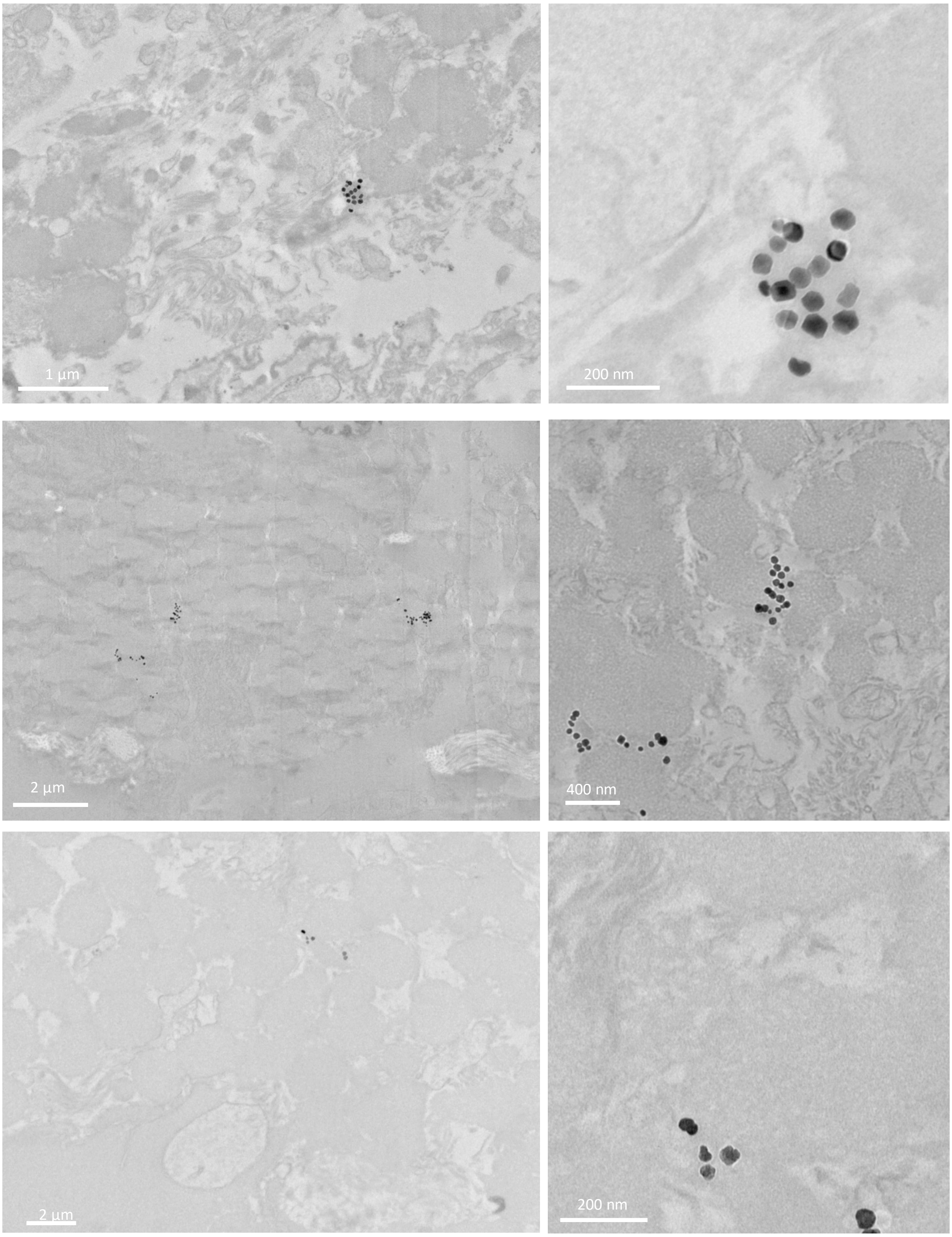
1 µm
200 nm
2 µm
400 nm
2 µm
200 nm

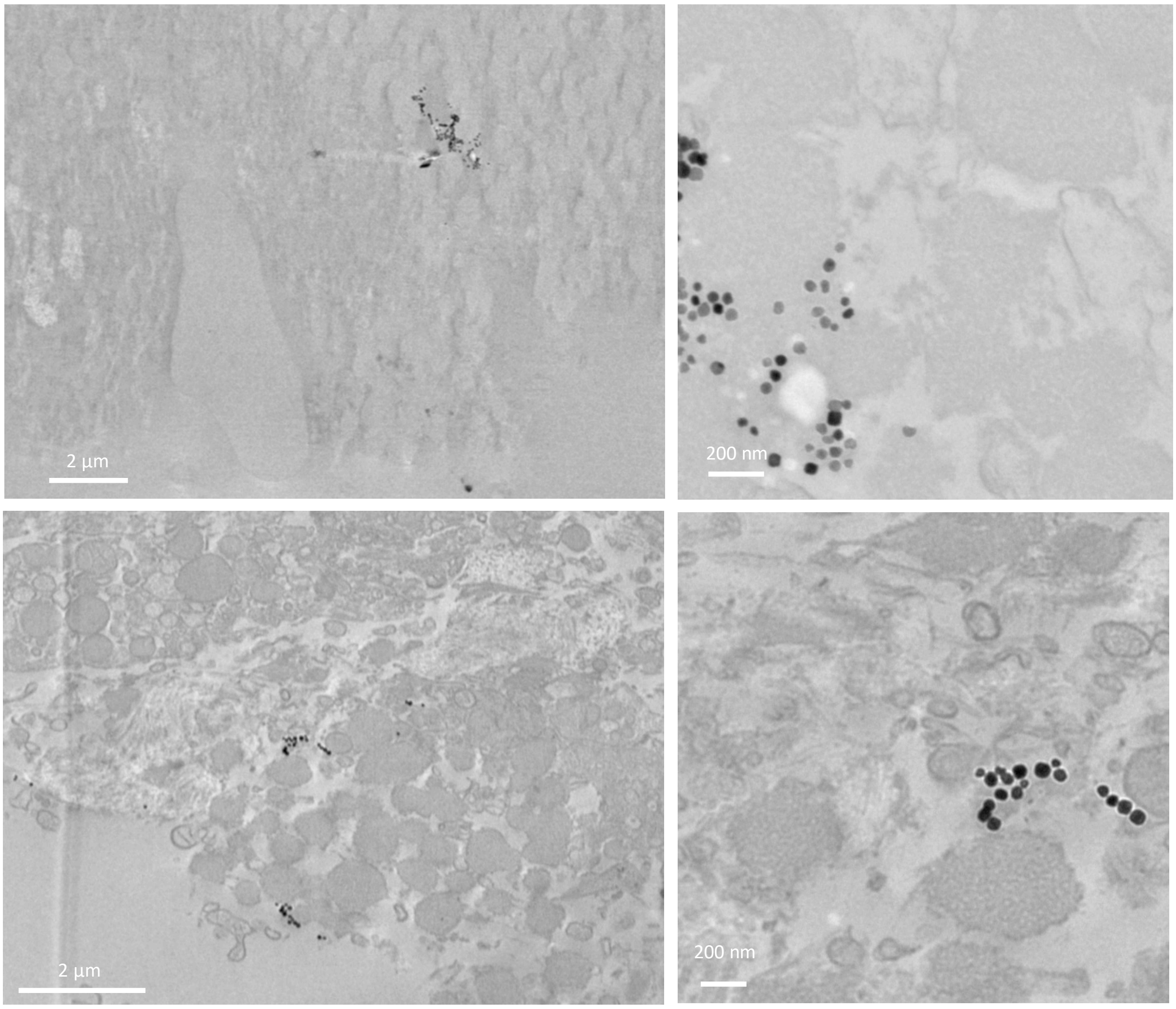

Figure 7. Transmission electron microscope imaging of orthotopic tumor models after intravenous administration of magnetosomes and upon magnet application.

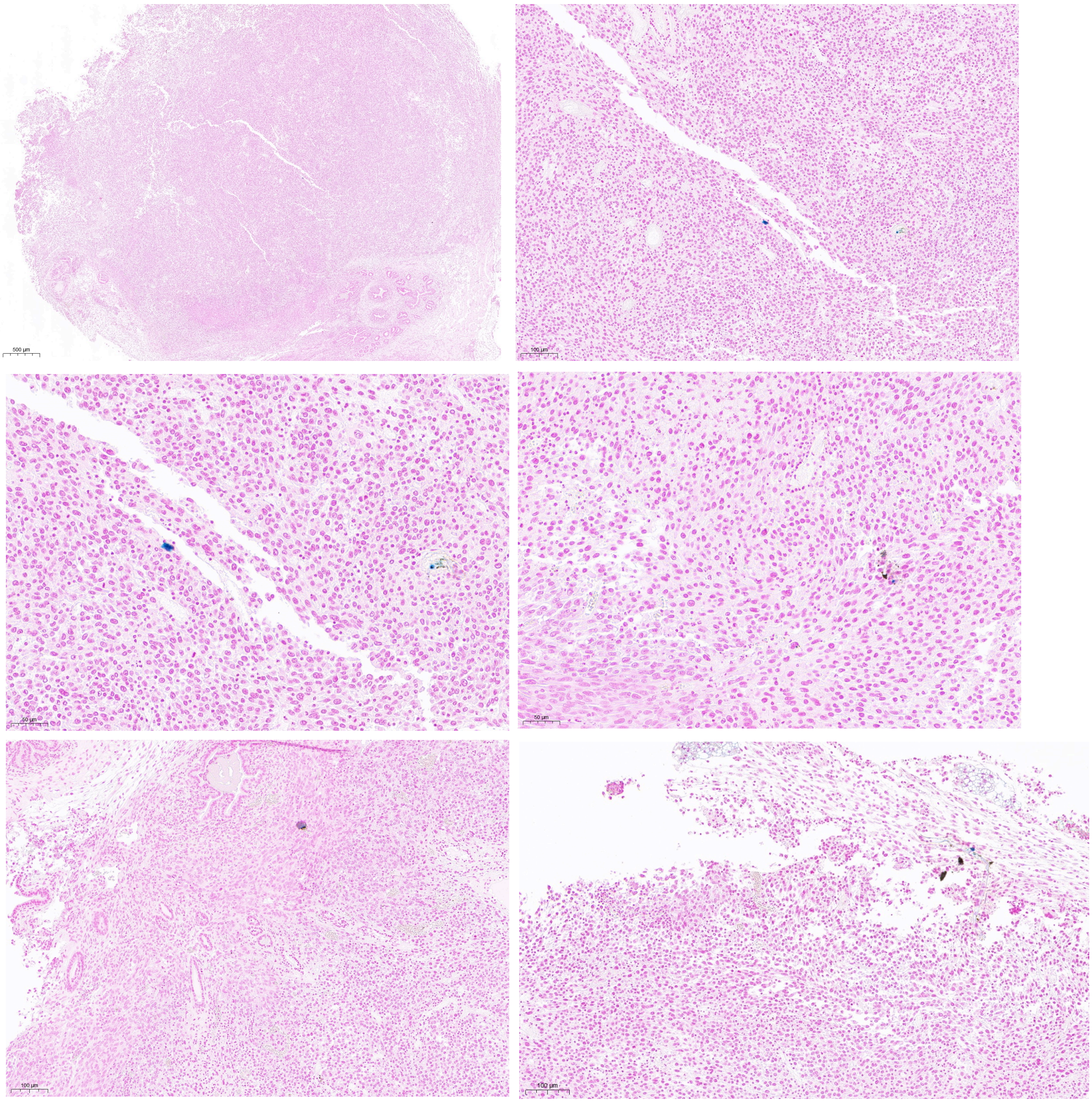

Figure 8. Histological Prussian Blue tissue staining in orthotopic tumor models after intravenous administration of magnetosomes and upon magnet application.

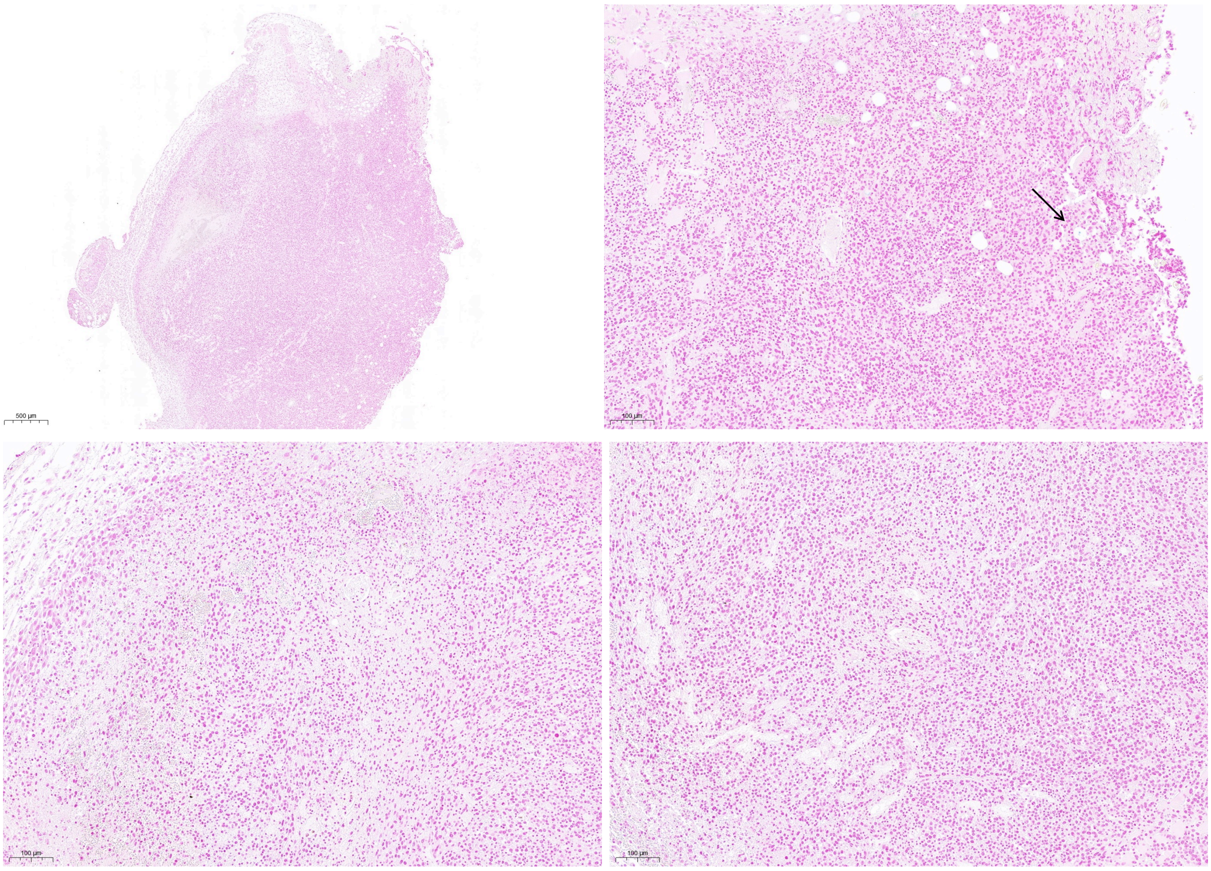

Figure 9. Histological Prussian Blue tissue staining in subcutaneous tumor models after intravenous administration of magnetosomes and without magnet application.

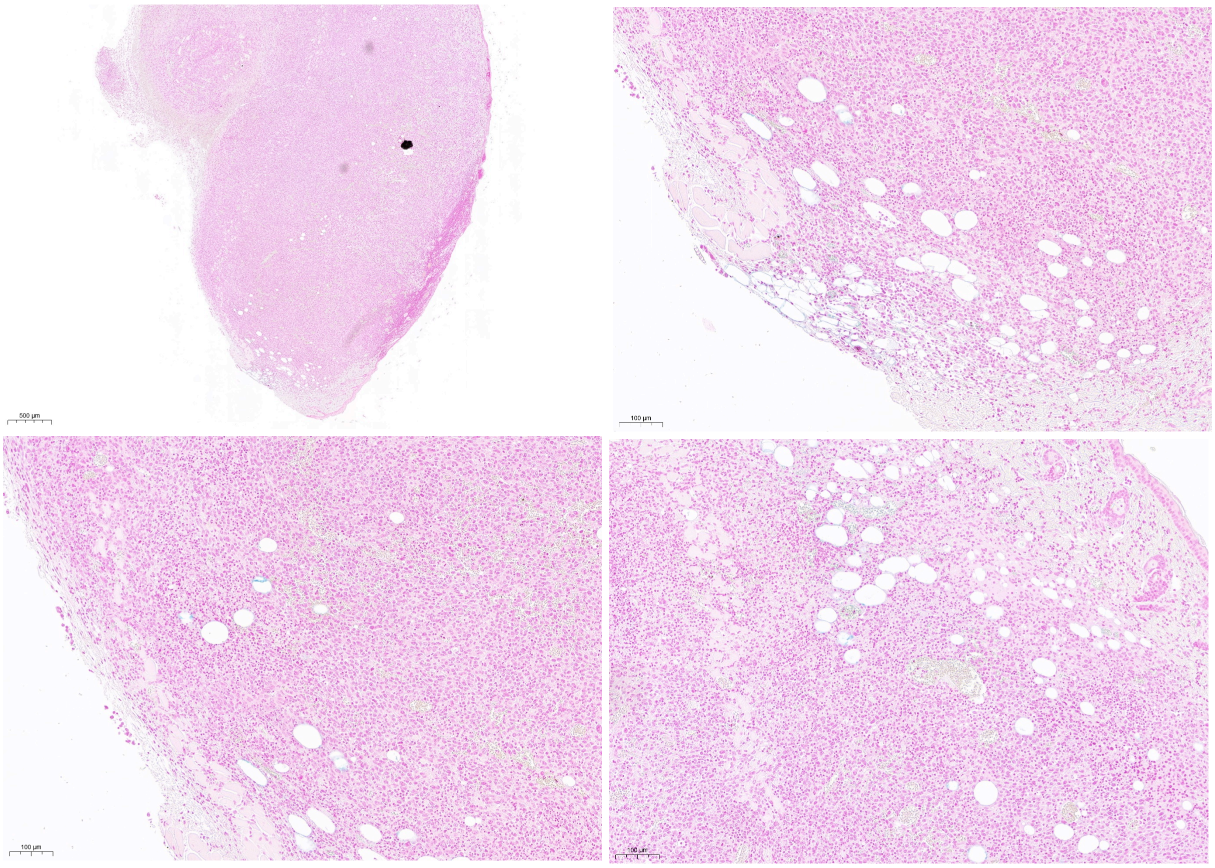

Figure 10. Histological Prussian Blue tissue staining in orthotopic tumor models after intravenous administration of magnetosomes and without magnet application.

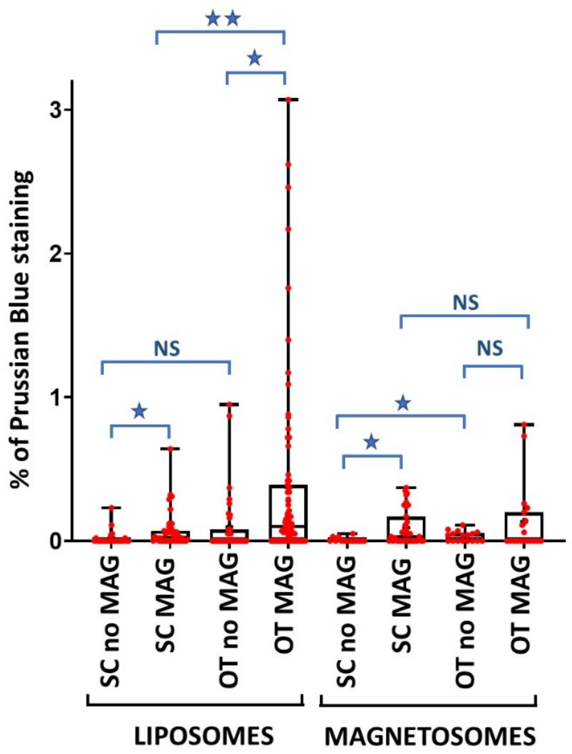

Figure 11: Prussian Blue quantification of nanoparticle presence in SC and OT tumor models with and without magnetic targeting. Quantification was done on whole histological tumor section image areas of 78400 µm² (320 µm x 245 µm). For each image, the Prussian Blue area was measured and divided by the total image area, providing the % of occupied area by the nanoparticles. Each measurement is shown as an independent red circle in the graph. **p* < 0.05, ***p* < 0.01, ****p* < 0.001 and NS (non-significant) for *p* > 0.05.

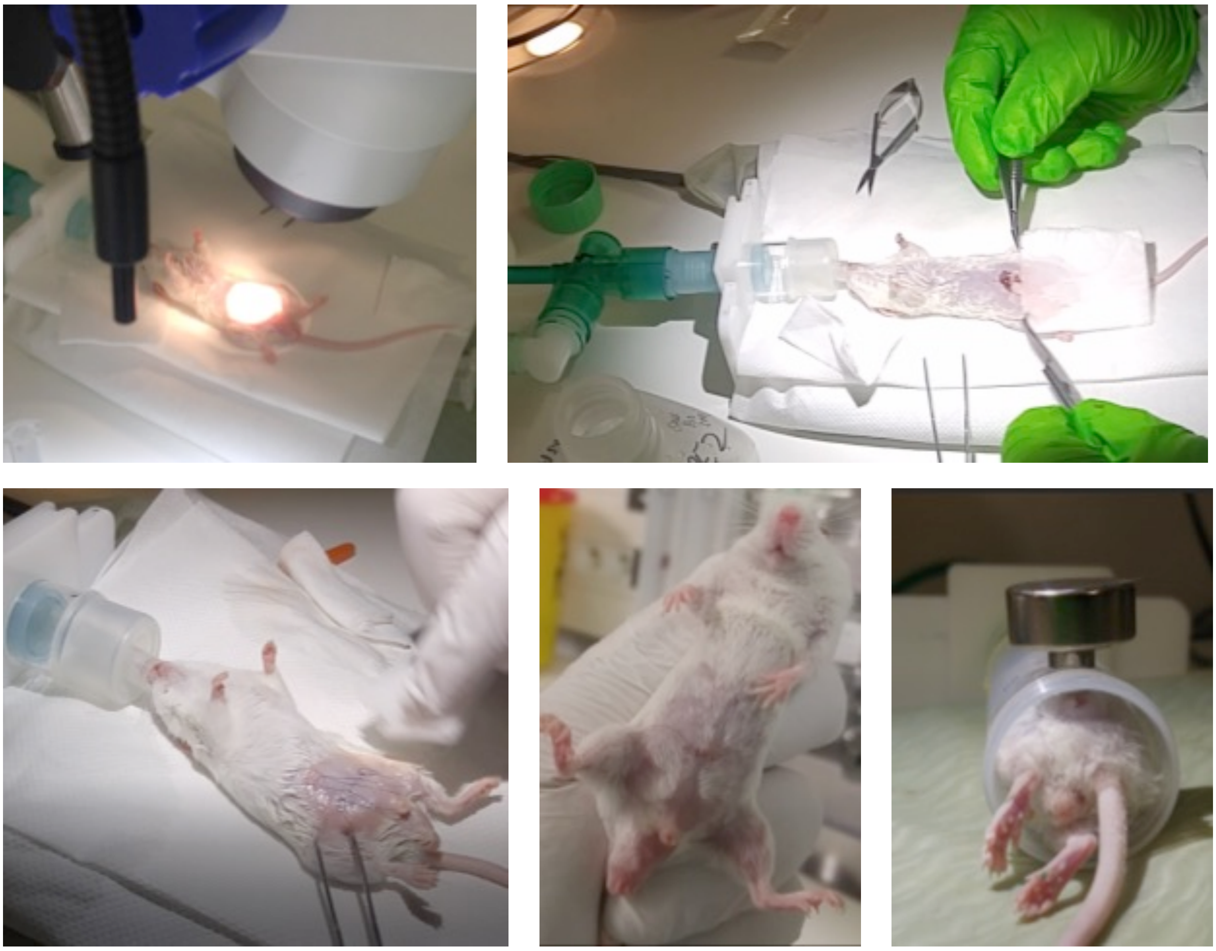

Figure 12. Microsurgery procedure for the cell injection within the prostate in order to generate the orthotopic prostate model. Bottom right image shows the magnet application procedure followed for tumor targeting of both the ultramagnetic liposomes and magnetosomes nanoparticles.
